# Genetic markers for the identification and characterization of *Opisthorchis viverrini*, a medically important food borne trematode in Southeast Asia

**DOI:** 10.1016/j.actatropica.2006.11.001

**Published:** 2006-12

**Authors:** Weerachai Saijuntha, Paiboon Sithithaworn, Sopit Wongkham, Thewarach Laha, Vichit Pipitgool, Trevor N Petney, Ross H Andrews

**Affiliations:** a Department of Biochemistry, Faculty of Medicine, Khon Kaen University, Khon Kaen 40002, Thailand; b Department of Parasitology, Faculty of Medicine, Khon Kaen University, Khon Kaen 40002, Thailand; c Institute of Zoology 1: Ecology and Parasitology, University of Karlsruhe, Kornblumen Strasse 13, Karlsruhe, Germany; d School of Pharmacy and Medical Sciences, University of South Australia, GPO Box 2471, Adelaide, SA 5001, Australia; e Liver Fluke and Cholangiocarcinoma Research Center (LFCRC), Faculty of Medicine, Khon Kaen University, Khon Kaen 40002, Thailand

**Keywords:** *Opisthorchis viverrini*, Genetic variation, Multilocus enzyme electrophoresis

## Abstract

The liver fluke, *Opisthorchis viverrini*, is one of the major food borne trematodes in Southeast Asia, where infection causes hepatobiliary disease and subsequent development of cholangiocarcinoma. In Thailand, *O. viverrini* is most prevalent in the northeast where there is marked regional variation in the rate of infection in humans at provincial, district and village levels. To date, the roles of genetic variation of *O. viverrini* on this observed variability in infection, transmission and associated disease are not known. We have applied multilocus enzyme electrophoresis (MEE), specifically allozyme electrophoresis, to isolates of *O. viverrini* from Thailand and Laos to establish genetic markers to examine its systematics and population structure. Forty-six enzymes commonly found useful for genetic characterisation in parasitic helminths were screened, and of these, 33 enzymes gave sufficient staining and resolution to act as potential genetic markers. Sixteen enzymes were monomorphic and 17 enzymes were polymorphic in the pools of worms examined. Whether they are indicative of different enzyme loci, heterozygosity or unique genotypes within the pools of worms examined remains to be determined. Preliminary investigations examining five individual worms at enzyme loci where pools of worms showed multiple bands have confirmed the diagnostic value of the enzyme loci established as well as providing evidence of potential population sub structuring and heterozygosity. For the first time, we have established at least 17 enzymes that provide the basis to undertake comprehensive genetic analyses of the systematics and population structure of *O. viverrini*, a medically important food borne trematode in Southeast Asia.

The liver fluke, *Opisthorchis viverrini*, is endemic in Thailand, Laos PDR, Cambodia and Vietnam (WHO, 1995). The most serious consequence of liver fluke infection is the development of cholangiocarcinoma, which is a cancer of the bile duct (Vatanasapt et al., 1990; IARC, 1994). Although the average prevalence of *O. viverrini* infection in Thailand is 9.4% (Jongsuksuntigul, 2002), there is a marked geographical variation. Within the northeast region where *O. viverrini* is most prevalent (Preuksaraj, 1984; Jongsuksuntigul, 2002), high variability of prevalence and intensity of infection has been documented at the provincial, district and village levels (Sithithaworn and Haswell-Elkins, 2003). Currently there is no information on the role of genetic variation on geographical distribution of *O. viverrini*. Previous studies on cox1 mitochondrial DNA (Ando et al., 2001) and RAPD analyses (Sithithaworn et al., in press) have detected molecular variation between isolates of *O. viverrini* from Thailand and Laos, suggesting potential population sub-structuring.

Multilocus enzyme electrophoresis (MEE) has been shown to be a powerful technique to examine the systematics and population structure of many vertebrate and invertebrate organisms, including, parasites, e.g. *Schistosome* (He et al., 1991; Chilton et al., 1999), *Echinococcus* (Lymbery and Thompson, 1988), *Fasciola* (Agatsuma et al., 1994), *Paragonimus* (Agatsuma and Habe, 1986). The usefulness of the application of MEE to provide answers to parasite systematics has been reviewed by Andrews and Chilton (1999). It has received limited application, however, to food-borne trematodes. For instance, a recent study applied MEE to isolates of *Clonorchis sinensis*, a closely related species to *O. viverrini* (Park et al., 2000). Eight enzymes, presumed to be encoded by 10 loci, were established. Multiple banding patterns were detected at three enzyme loci and one enzyme had the potential to be a genetic marker between two geographically separate populations. The inherent dangers in using too few enzyme loci (i.e. genetic markers) in the identification and characterisation of parasites have been discussed in detail in Andrews and Chilton (1999).

This is the first study to apply MEE to establish sufficient genetic markers to enable appropriate comprehensive examinations of the systematics and population structure of *O. viverrini* and follows the guidelines detailed in Andrews and Chilton (1999).

Adult worms of *O. viverrini* were obtained from hamsters previously infected with metacercariae from naturally infected cyprinid fishes from four different geographical areas. Two isolates from Khon Kaen Province, Thailand, namely Ban Lerngpleuy (KLp) and Ban Phai (KBp), and two isolates from Laos, Nam Ngum (NG) and Vientiane (VT) provinces, were collected for analysis. Hamsters were infected with 50–100 metacercaria by intragastric intubations and were sacrificed at 4–6 months after infection. Adult worms were collected from hamster liver bile ducts, washed several times with cooled 0.85% NaCl and then kept in a microcentrifuge tube at −80 °C until all samples became available for analysis.

Twenty adult worms from KBp were pooled to achieve adequate enzyme concentrations to maximise the number of detectable enzymes and loci. Twenty-five microlitre of cold lysing solution were added and the worms were homogenized by sonication (‘Vibra cell’; Sonic and Meterials, USA). The homogenate was centrifuged at 10,000 rpm for 5 min at 4 °C and the supernatant was removed with a capillary tube and then kept at −20 °C (Richardson et al., 1986). Homogenates from individual worms were prepared by adding 5 μl of cold lysing solution and hand homogenizing each worm by grinding in a U-plate for application to the electrophoretic support medium.

Electrophoresis was conducted on cellulose acetate (‘Cellogel’; Chemitron, Milan) according to the methods described by Richardson et al. (1986). The current was kept constant at 200 V and the running time was adjusted between 1.5 and 3 h according to the mobility of each enzyme. The loading position of the sample was set at the cathode. In total, 46 enzymes commonly found in parasitic helminths were screened. Hamster liver homogenate was used as a positive control of the enzymic activity as well as acting as a control to ensure that only liver fluke enzymes were scored.

The 46 enzymes screened were assayed in different conditions, running time and running buffer as showed in Table 1. Thirty-three of the 46 enzymes screened from pools of *O. viverrini* adult worm extract showed sufficient staining intensity and resolution to enable genetic interpretation. Each enzyme exhibited between 1 and 5 bands following histochemical staining. Sixteen enzymes (ACP, ALD, EST, FDP, G6PD, GPI, HBDH, IDH, NP, PEP-B, PEP-C, PEP-D, PGAM, 6PGD, PK and UMPK) were monomorphic (one band). Six enzymes had two bands (CK, FUM, GDH, LDH, ME and PEP-A) whereas eight enzymes (ACON, AK, ENOL, GOT, GPT, HK, MDH and PGM) showed three bands. Only one enzyme (NDPK) showed four bands, whereas two enzymes (GAPD and TPI) exhibited as many as five bands (Table 1). Of the 16 monomorphic enzymes, sharp bands were detected following histochemical staining of eleven enzymes which provided no interpretation problems, whereas at five enzymes (FDP, G6PD, IDH, PGAM and PK) single broad bands were detected (Table 1).

To determine the significance of these banding patterns following the MEE analysis of samples of pools of individuals we compared individual worms with the pools using the rationale and allozyme interpretation as detailed by Andrews and Chilton (1999). Ten individual worms from KBp and 10 from NG were used to examine multiple banding patterns and single broad bands detected at specific enzymes following electrophoresis of pools of worms. Sufficient homogenate was available from an individual worm to examine up to 10 enzymes, which resulted in adequate intensity and resolution following histochemical staining to enable genetic interpretation. The following enzymes, ENOL, FDP, GAPD, GOT, G6PD, IDH, PGAM, PGM, PK and TPI were chosen for comparison between four isolates because they showed the range of multiple banding patterns (including single broad bands) detected in pools of worms following MEE analysis which could reflect the presence of multiple enzyme loci and/or mixtures of different genotypes.

Comparisons of pools and individual worms showed that for the 10 enzymes examined the multiple bands detected in pools were not due to multiple loci. Rather, they showed the presence of mixes of homozygous and heterozygous patterns at these loci when quaternary structure is taken into account. For instance, the broad bands detected in pools at FDP, G6PD, IDH, PGAM and PK was shown to represent a mix of different homozygous genotypes. Analysis of individual worms confirmed the diagnostic value of IDH, which was shown to distinguish pools of worms from Khon Kaen and Laos. Interestingly, comparison of pools and individual worms confirmed the diagnostic potential of G6PD to distinguish worms from KBp in Khon Kaen versus KLp and KBp versus the two isolates from Laos. On the other hand, the enzyme PK, which resolved as a broad band and was potentially diagnostic when pools were examined (i.e. KBp versus KLp and the Khon Kaen isolates versus Laos isolates, Table 2), contains an individual worm from NG that shares allele *b* with the Khon Kane isolates, hence reducing it's diagnostic capabilities. Nevertheless, the enzyme PK is diagnostic when individuals and pools from VT (allele *c*) versus Khon Kane isolates (alleles *a* and *b*) are compared.

Following analysis of individual worms for the enzymes ENOL, GAPD, GOT, PGM and TPI, where multiple bands were detected in pools, results showed that they represented single banded homozygous and multiple banded heterozygous genotypes. For instance, at the dimeric enzymes ENOL, GAPD, GOT and TPI individual worms showed single banded homozygous and the typical three banded heterozygous patterns, whereas at the monomeric enzyme, PGM, typical double banded heterozygous and single banded homozygous patterns were detected. Comparisons of individual worms and pools from the four localities confirmed the presence of heterozygous and homozygous individuals at the dimeric enzyme ENOL as well as confirming the diagnostic value of this enzyme where isolates from Khon Kaen and Laos have unique alleles (*b* and *d* versus *a* and *c*, respectively).

In general, in order to measure the extent of genetic divergence between populations and species and hence determine the biological significance of such divergence, it is important to use as many enzyme loci (i.e. independent characters/markers) as possible. For example, Andrews and Chilton (1999) have recommended that at least 15 enzyme loci should be used to confidently differentiate between strains of parasite. Previous studies of the systematics of trematodes have utilised from 10 to 16 enzyme loci in *C. sinensis* (Park et al., 2000), *S. japonicum* (Chilton et al., 1999) and *Fasciola* sp. (Agatsuma et al., 1994). Although there are inherent dangers in comparing across different electrophoresis studies (Andrews and Chilton, 1999; Richardson et al., 1986), seven of the eight enzymes previously established for the closely related species, *C. sinensis*, were also detected in *O. viverrini*, while one enzyme (GPD) was not detectable in *O. viverrini*. We detected three bands in two enzymes (ACON, MDH) in *O. viverrini* while two bands were described in *C. sisnensis* (Park et al., 2000).

Using cellulose acetate as the electrophoresis support medium, as in our study, has significant advantages because it allows the analysis of samples where only small volumes are available, such as individual *O. viverrini* worms. In our study, worms were pooled to increase the volume of sample available for analysis thereby maximising the number of potential enzymes that can be available for future genetic analyses. For the first time, we have established 33 enzymes that can now be used to conduct comprehensive analyses of the systematics of the medically significant food borne trematode *O. viverrini.* A comprehensive analysis of individual worms compared to the pools of worms analysed herein is required to determine the significance of the multiple banding patterns that have been detected in the pools.

We have shown that the homogenate from one individual worm provides sufficient sample for the analysis of 10 enzymes using cellulose acetate as the support medium. It is heartening that our preliminary investigations using five individual worms available for comparisons with pools of worms from two localities in Thailand and two localities in Laos confirms that the 10 enzymes which showed either multiple bands or broad single bands following MEE analyses of pools have indeed diagnostic value. For example, three of these enzymes (ENOL, G6PD and IDH) can now be used as diagnostic markers and all 10 enzymes together with the 16 monomorphic enzymes expressed in pools can be now be used in analyses of the systematics and population structure of *O. viverrini*. Further investigations of individual worms are required to confirm the diagnostic potential of the remaining 12 enzymes that showed multiple bands in pools. Nonetheless, our results agree with a previous study of Ando et al. (2001) who found intraspecific variation between isolates from northeast Thailand using the mitochondrial cox1 sequence.

The enzyme markers that we have established now provide the basis to conduct comprehensive allozyme analyses of *O. viverrini* isolates (both pools and individuals where appropriate) from different geographical localities to provide a better understanding of the systematics and population structure of this medically important liver fluke in Southeast Asia. These investigations have significant implications concerning the implementation and establishment of effective control and surveillance programs targeted to medically important food-borne parasites.

## Figures and Tables

**Table 1:** A list of 46 enzymes examined in *O. viverrini* by MEE under different experimental conditions, including the number of bands of activity exhibited per enzyme following staining

Enzymes (abbreviation, enzyme commission no.)	Running buffer^a^	Running time (h)
Enzymes (one band)		
Acid phosphatase (ACP, 3.1.3.2)	B	2.30
Aldolase (ALD, 4.1.2.13)	B	2.00
Esterase (EST, 3.1.1.1)	B	2.00
Fructose-1,6-diphosphatase (FDP, 3.1.3.11)^b^	B	2.30
Glucose-6-phosphate dehydrogenase (G6PD, 1.1.1.49)^b^	B, C	2.30
Glucose-phosphate isomerase (GPI, 5.3.1.9)	B, C	2.30
*β*-Hydroxybutyrate dehydrogenase (HBDH, 1.1.1.30)	B	1.30
Isocitrate dehydrogenase (IDH, 1.1.1.42)^b^	A, B	2.30
Nucleoside phosphorylase (NP, 2.4.2.1)	B	1.30
Peptidase leucine-glycine-glycine (PEP-B, 3.4.11.4)	B	2.30
Peptidase lysine-leucine (PEP-C, 3.4.13)	B	2.30
Peptidase phenylalanine-proline (PEP-D, 3.4.13)	B, C	2.00
Phosphoglycerate mutase (PGAM, 2.7.5.3)^b^	C	2.30
6-Phosphogluconate dehydrogenase (6PGD, 1.1.1.44)	B	1.30
Pyruvate kinase (PK, 2.7.1.40)^b^	C	2.30
Uridine monophosphate kinase (UMPK, 2.7.1.48)	C	2.30

Enzyme (two bands)		
Creatine kinase (CK, 2.7.3.2)	B, C	2.30
Fumarate hydratase (FUM, 4.2.1.2)	B	2.30
Glutamate dehydrogenase (GDH, 1.4.1.3)	B	1.30
Lactate dehydrogenase (LDH, 1.1.1.27)	B	1.30
Malic enzyme (ME, 1.1.1.40)	B, C	2.30
Purine peptidase valine-leucine (PEP-A, 3.4.13.11)	B, C	2.00

Enzymes (three bands)		
Aconitate hydratase (ACON, 4.2.1.3)	B	1.30
Adenylate kinase (AK, 2.7.4.3)	B	2.30
Alanine aminotransferase (GPT, 2.6.1.2)	B	2.00
Aspartate aminotransferase (GOT, 2.6.1.1)	B	2.00
Enolase (ENOL, 4.2.1.11)	A, B	2.00
Hexokinase (HK, 2.7.1.1)	B, C	2.30
Malate dehydrogenase (MDH, 1.1.1.37)	B, C	2.30
Phosphoglucomutase (PGM, 2.7.5.1)	B, C	2.30

Enzyme (four bands)		
Nucleotide diphosphate kinase (NDPK, 2.7.4.6)	B	2.30

Enzymes (five bands)		
Glyceraldehyde-3-phosphate dehydrogenase (GAPD, 1.2.1.12)	B	2.30
Triose-phosphate isomerase (TPI, 5.3.1.1)	B	2.00

a A = 0.01 M Citrate-phosphate pH 6.4, B = 0.02 M Phosphate pH 7.0, C = 0.05 M Tris-maleate pH 7.8.

b Enzymes showed one broad band. The following enzymes showed no activity following electrophoresis and staining: alcohol dehydrogenase (ADH, 1.1.1.1), aldehyde dehydrogenase (ALDH, 1.2.1.5), alkaline phosphatase (AP, 3.1.3.1), diaphorase (DIA, 1.6.*.*), glucose dehydrogenase (GLDH, 1.1.1.47), glycollate oxidase (GOX, 1.1.3.1), glycerol-3-phosphate dehydrogenase (GPD, 1.1.1.8), leucine aminotransferase (LAP, 3.4.11.1), mannose-phosphate isomerase (MPI, 5.3.1.8), phosphoglycerate kinase (PGK, 2.7.2.3), superoxide dismutase (SOD, 1.15.1.1), sorbital dehydrogenase (SORDH, 1.1.1.14) and xanthine oxidase (XO, 1.2.3.2).

**Table 2:** Allelic profile of pooled and five individual worms of four different geographical isolates at 10 enzyme loci

Isolate^a^	Worm^b^	Enol	Gapd	Got	Pgm	Tpi	Fdp	G6pd	Idh	Pgam	Pk
KLp	Pools	b, c, d	a, b, c, d, e	a, b, c	a, b, c	a, b, c, d, e	A^c^	B	B	A	B
	1	bd	ac	a	a	c	b	c	b	b	b
	2	b	ae	a	b	a	a	d	b	b	b
	3	b	ac	a	ab	c	b	d	b	b	b
	4	b	a	a	a	ce	a	d	b	c	b
	5	b	ae	a	a	c	b	c	b	c	b

KBp	Pools	b, c, d	a, b, c, d, e	a, b, c	a, b, c	a, b, c, d, e	A	A	B	A	A
	1	bd	ac	a	a	c	a	b	b	b	a
	2	b	ac	a	ab	c	b	a	b	c	a
	3	b	ae	c	a	ac	c	b	b	b	b
	4	b	ae	a	ab	ac	a	a	b	b	b
	5	bd	ae	a	ab	ac	a	b	b	c	b

NG	Pools	a, b, c	a, b, c, d, e	a, b, c	a, b, c	a, b, c, d, e	A	B	A	A	C
	1	a	ae	a	ac	ac	b	d	a	c	b
	2	a	ac	ac	a	c	b	d	a	a	c
	3	a	ac	a	ac	ce	a	c	a	c	c
	4	a	ac	a	a	ac	a	d	a	c	c
	5	a	ae	ac	ac	c	b	c	a	c	c

VT	Pools	a, b, c	a, b, c, d, e	a, b, c	a, b, c	a, b, c, d, e	A	B	A	A	C
	1	a	ae	a	a	a	b	c	a	c	c
	2	a	ae	a	a	ac	b	d	a	c	c
	3	a	ac	a	ac	ac	b	c	a	b	c
	4	ac	ac	a	a	ae	b	c	a	c	c
	5	ac	ae	ac	ab	ac	a	c	a	a	c

a KLp = Ban Lerngpleuy, Khon Kaen Province, Thailand; KBp = Ban Phai, Khon Kaen Province, Thailand; NG = Nam Ngum Province, Laos; VT = Vientiane Province, Laos.

b Pools = pools of 20 worms; 1–5 = each of five individual worms.

c A, B and C represented single broad band.
